# Towards a molecular understanding of cellulose dissolution in ionic liquids: anion/cation effect, synergistic mechanism and physicochemical aspects

**DOI:** 10.1039/c7sc05392d

**Published:** 2018-03-26

**Authors:** Yao Li, Jianji Wang, Xiaomin Liu, Suojiang Zhang

**Affiliations:** a Beijing Key Laboratory of Ionic Liquids Clean Process , CAS Key Laboratory of Green Process and Engineering , Institute of Process Engineering , Chinese Academy of Sciences , Beijing , 100190 , P. R. China . Email: xmliu@ipe.ac.cn ; Email: sjzhang@ipe.ac.cn; b Collaborative Innovation Center of Henan Province for Green Manufacturing of Fine Chemicals , School of Chemistry and Chemical Engineering , Key Laboratory of Green Chemical Media and Reactions , Henan Normal University , Xinxiang , Henan 453007 , P. R. China

## Abstract

Cellulose is one of the most abundant bio-renewable materials on the earth and its conversion to biofuels provides an appealing way to satisfy the increasing global energy demand. However, before carrying out the process of enzymolysis to glucose or polysaccharides, cellulose needs to be pretreated to overcome its recalcitrance. In recent years, a variety of ionic liquids (ILs) have been found to be effective solvents for cellulose, providing a new, feasible pretreatment strategy. A lot of experimental and computational studies have been carried out to investigate the dissolution mechanism. However, many details are not fully understood, which highlights the necessity to overview the current knowledge of cellulose dissolution and identify the research trend in the future. This perspective summarizes the mechanistic studies and microscopic insights of cellulose dissolution in ILs. Recent investigations of the synergistic effect of cations/anions and the distinctive structural changes of cellulose microfibril in ILs are also reviewed. Besides, understanding the factors controlling the dissolution process, such as the structure of anions/cations, viscosity of ILs, pretreatment temperature, heating rate, *etc.*, has been discussed from a structural and physicochemical viewpoint. At the end, the existing problems are discussed and future prospects are given. We hope this article would be helpful for deeper understanding of the cellulose dissolution process in ILs and the rational design of more efficient and recyclable ILs.

## Abstract 2

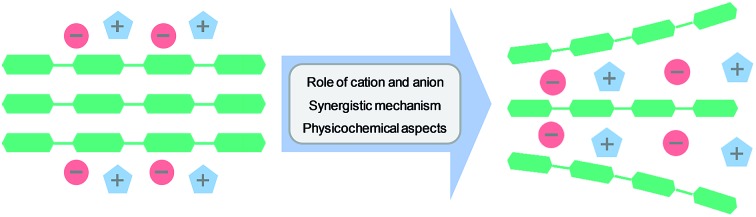
This perspective summarizes mechanistic studies on cellulose dissolution in ionic liquids, highlighting the synergistic mechanism, physicochemical aspects and future research trends.

## 1. Introduction

Lignocellulosic biomass is the most abundant renewable raw material on the earth, with an estimated global production of around 1.1 × 10^11^ tons per year.^1^ The three major constituents of lignocellulosic biomass are cellulose (40–50 wt%), hemicellulose (25 wt%) and lignin (25 wt%),^2^,^3^ so that there are 40 billion tons of cellulose renewed annually.^4^ According to a recent report,^5^ only 0.1 billion tons of cellulose are used as feedstock for industry, so there is a huge amount of undeveloped value in cellulose.

**Fig. 1:**
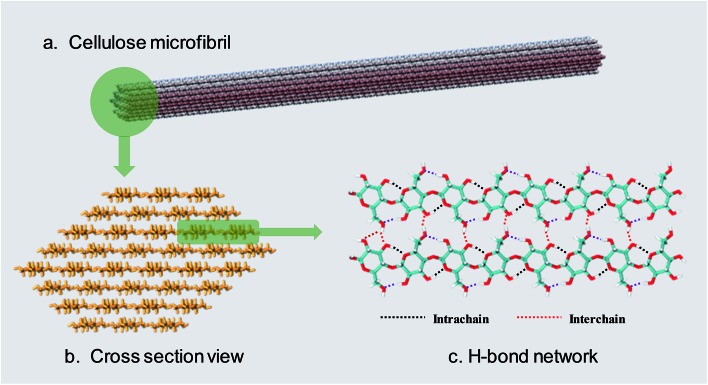
(a) The usual form of existence of cellulose–cellulose microfibril. (b) Cross-section view of a 36 chain cellulose elementary microfibril. (c) The H-bond network between cellulose chains.

Cellulose is a linear polysaccharide consisting of numerous d-glucose units linked through β(1–4) glycosidic bonds.^6^ As shown in Fig. 1a and b, the natural form of cellulose is a microfibril structure,^7^ where chains align parallel to form flat sheets, and the sheets stack together to form a three-dimensional crystal structure with a wide range of diameters (2–20 nm) and lengths (0.1–100 μm).^8^,^9^ The crystal structure has a complex hydrogen bond (H-bond) network. O–H···O H-bonds are formed by the nearby hydroxyl groups of neighbouring glucose units in the same chain (intrachain) or different chains (interchain).^10^–^12^ Besides, a lot of van der Waals (vdW) interactions connect residues on contiguous sheets (intersheet),^13^–^15^ which give rise to the strength and robustness of cellulose crystals. The intrachain, interchain and intersheet interactions provide sufficient strength against deconstruction^14^,^16^,^17^ and therefore cellulose is not soluble in water or other conventional organic solvents.^18^ The highly ordered structure and interactions also make it difficult to deconstruct cellulose polymers to monomers, which is termed “recalcitrance”.^14^

Dissolution is a necessary pretreatment step to overcome the recalcitrance before conversion of cellulose to value-added chemicals.^19^ Traditional nonaqueous and aqueous solvent systems for cellulose, such as sodium hydroxide/carbon disulfide mixtures,^20^*N*-methylmorpholine oxide (NMMO),^21^ dimethyl sulfoxide (DMSO)/tetrabutylammonium fluoride (TBAF),^22^ and aqueous solutions of metal complexes,^23^ suffer drawbacks, like high energy cost, volatility, toxicity, poisonous gas pollution, poor solvent recovery, or insufficient solvation power. Therefore, there is an increasing demand to develop green alternative solvents to overcome these defects.

Ionic liquids (ILs) refer to liquid salts composed of ions with melting temperature around or below 100 °C.^24^ The most common examples of ILs include salts with organic cations (alkylimidazolium [R_1_R_2_IM]^+^, alkylpyridinium [RPy]^+^, tetraalkylammonium [NR_4_]^+^, or tetraalkylphosphonium [PR_4_]^+^), and inorganic anions (hexafluorophosphate [PF_6_]^–^, tetrafluoroborate [BF_4_]^–^, and several low melting chlorides, bromides, and iodides). ILs present a lot of advantages compared with traditional solvents, such as high thermal stability, wide electrochemical window, wide liquid range, low vapor pressure and high solvation ability towards various chemical substances, which leads to their widespread applications in a variety of fields.^24^ The physical and chemical properties of ILs can be regulated by altering the structures of cations and anions for different purposes.^25^ In 2002, Swatloski *et al.*^26^ reported that cellulose could be dissolved in 1-butyl-3-methylimidazolium chloride ([Bmim][Cl]) without derivatization. Since then, the applications of ILs in cellulose pretreatment were carried out and many kinds of effective ILs have been reported. Generally speaking, the most efficient anions are [Cl]^–^, [OAc]^–^, [HCOO]^–^ and [(EtO)_2_PO_2_]^–^ while non-coordinating anions such as [BF_4_]^–^ and [PF_6_]^–^ cannot dissolve cellulose. ILs with imidazolium, pyridinium, ammonium, and phosphonium cations are often better cellulose solvents and all have unsaturated aromatic structures in their cations.^27^ Moreover, several aprotic co-solvents, such as dimethyl sulfoxide (DMSO) and *N*,*N*-dimethylformamide (DMF), can enhance the dissolution of cellulose in ILs.^28^,^29^

The typical pretreatment process based on IL solvents includes the following steps: dissolution–recovery–desiccation.^30^,^31^ Among these steps, dissolution is of great importance because during dissolution the crystallographic form of cellulose can be transformed to cellulose II, which has a much faster enzyme hydrolysis rate in the subsequent conversion step. Understanding why ILs can dissolve cellulose is essential for the design of more efficient and biocompatible ILs as well as optimization of the existing pretreatment technologies. Therefore, a large number of papers have been published to gain knowledge on the dissolution mechanism, and it is generally believed that H-bond formation between anions and the hydroxyl groups of cellulose is the main reason for cellulose dissolution in ILs.^32^ However, certain things are still unclear and controversial, such as the role of the cation, the contribution of hydrophobic interaction and the microscopic dissolution process.^33^,^34^ Considering the rapid development of IL-based cellulosic biomass processing, it is necessary to overview previous studies relevant to the understanding of cellulose dissolution in ILs, with a summary of specific behaviors and governing factors in the process.

In this perspective, we begin with a brief overview of cellulose dissolution in ILs, including the developmental history of ILs and cellulose solubility order of typical ILs, followed by a summary of the experimental mechanistic studies. Then simulation studies with different computational methods are introduced. After that, recent progress in the investigation of the dissolution mechanism of cellulose in ILs is reviewed, with deduction of the synergistic mechanism, and the factors affecting cellulose dissolution are also discussed. At the end of the article, a brief conclusion and future prospects have been presented.

## 2. A brief overview of cellulose dissolution in ILs

**Fig. 2:**
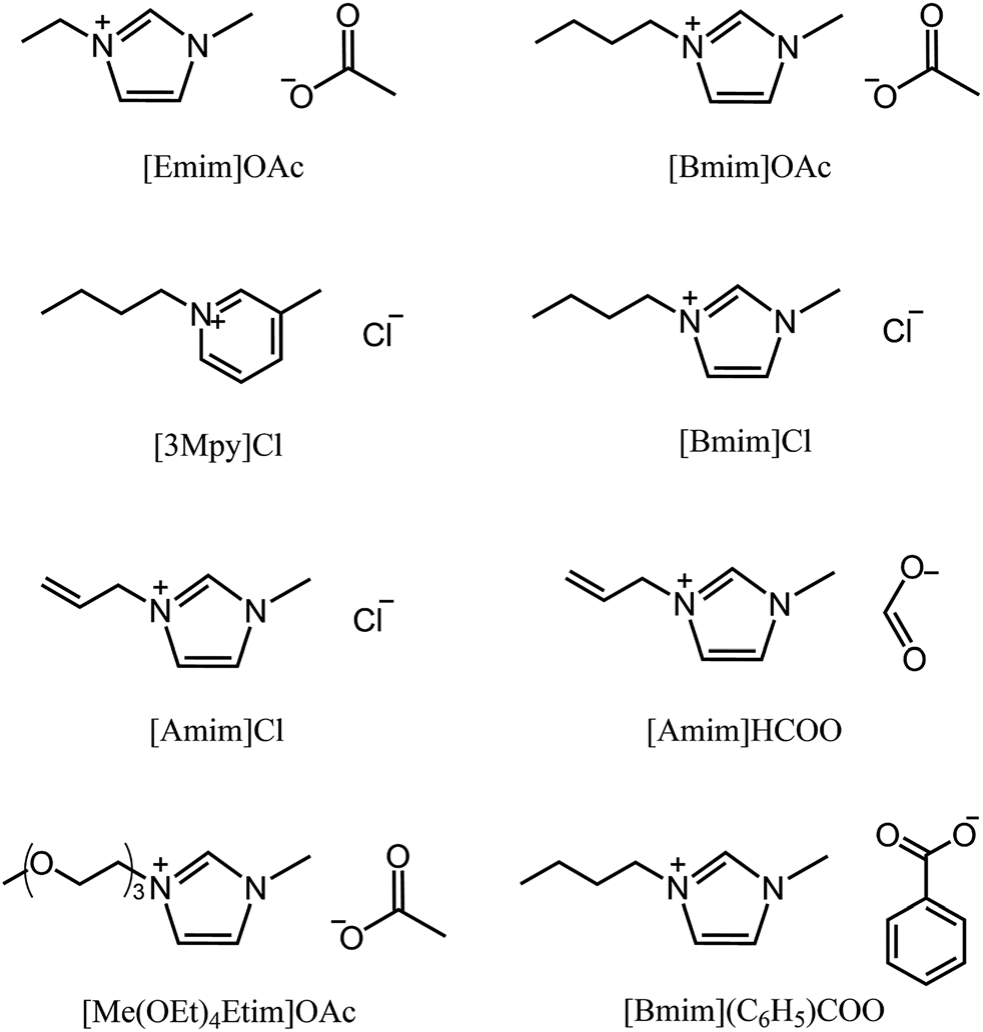
Commonly used ILs with good cellulose solubility.

The earliest report on the dissolution of cellulose using ILs can be dated back to 1934, when Graenacher patented that *N*-alkylpyridinium chloride was capable of dissolving cellulose.^35^ Unfortunately, this discovery did not get much attention until the high dissolving ability of [Bmim][Cl] was reported by Rogers *et al.*^26^ in 2002. After this breakthrough, ILs with lower melting points and stronger dissolution ability, such as [Emim][OAc] and [Amim][Cl] were reported by Zhang *et al.*^36^ Heinze *et al.*^37^ pointed out that pyridinium based ILs were better cellulose solvents; for example, [Bpy][Cl] showed a solubility of cellulose as high as 37% at 105 °C. Köhler *et al.*^38^ claimed that when paired with formate anion, ILs with tributylmethyl-ammonium cation could dissolve certain amount of cellulose at 85 °C within 15 min. In 2006, Sixta *et al.*^39^ reported a new kind of acid–base conjugated ILs, prepared by combining several superbases (TMG, DBU) with organic acids, which were found to dissolve cellulose quickly and could be recycled efficiently with 99% purity after each recycle. A less toxic but more stable morpholinium IL, [AMMorp][OAc] was found by Raut *et al.*^40^ to dissolve cellulose at 30% mass fraction at 120 °C in 20 min. All the examples listed above indicate that a significant number of ILs can dissolve cellulose; Fig. 2 exhibits 8 kinds of commonly used ILs with a dissolving capacity of more than 30 g cellulose per mol of IL.^32^ Meanwhile, researchers introduced co-solvents into the IL–cellulose systems. Xu *et al.*^28^ found that by adding some aprotic solvent (DMSO, DMA and DMF) to [Bmim][OAc], the ability to dissolve cellulose could be greatly enhanced and the dissolution could be conducted at room temperature. Sun *et al.*^41^ used compressed CO_2_ as an anti-solvent and cellulose could be efficiently precipitated and refined from ILs, revealing a simple and cost-effective process. What's more, it was found that a small amount of water did not affect the dissolution capacity of cellulose in [Emim][OAc]^42^ and a mixed solvent of solid acid/[Bmim][Cl] could achieve better efficiency of cellulose conversion^43^ with easier recovery of sugars.

In general, ILs consisting of anions with weak H-bond basicity, such as [BF_4_]^–^, [PF_6_]^–^ and [Tf_2_N]^–^, were not effective solvents of cellulose. Thus it can be simply summarized from the experimental results that the dissolving capacity of ILs strongly depends on the H-bond acceptability of anions. Xu *et al.*^44^ showed that the H-bond accepting ability (*β* value) of the anions was closely linked to the solubility of cellulose and the *β* value obtained in the solvatochromatic study might be a good indicator of the ability to dissolve cellulose. On the other hand, the chemical structure of imidazolium cations also had a great influence on cellulose dissolution. When the chain length of alkyl groups or the symmetry of cations increased, the dissolution rate of cellulose in ILs decreased, because of the increase of viscosity and the decrease of H-bond acidity.^45^ In general, the dissolving ability of ILs with different cations follows the order of imidazolium-based ILs > pyridinium-based ILs > ammonium-based ILs, while the order for those with different anions is more specific, [OAc]^–^ > [HSCH_2_COO]^–^ > [HCOO]^–^ > [(C_6_H_5_)COO]^–^ > [H_2_NCH_2_COO]^–^ > [HOCH_2_COO]^–^ > [CH_3_CHOHCOO]^–^ > [DCA]^–^.^32^ It should be noted that this is only a general trend and the solubility depends on both the cationic and anionic structures of the ILs and other external conditions.

## 3. Experimental studies on the mechanism of cellulose dissolution in ILs

**Fig. 3:**
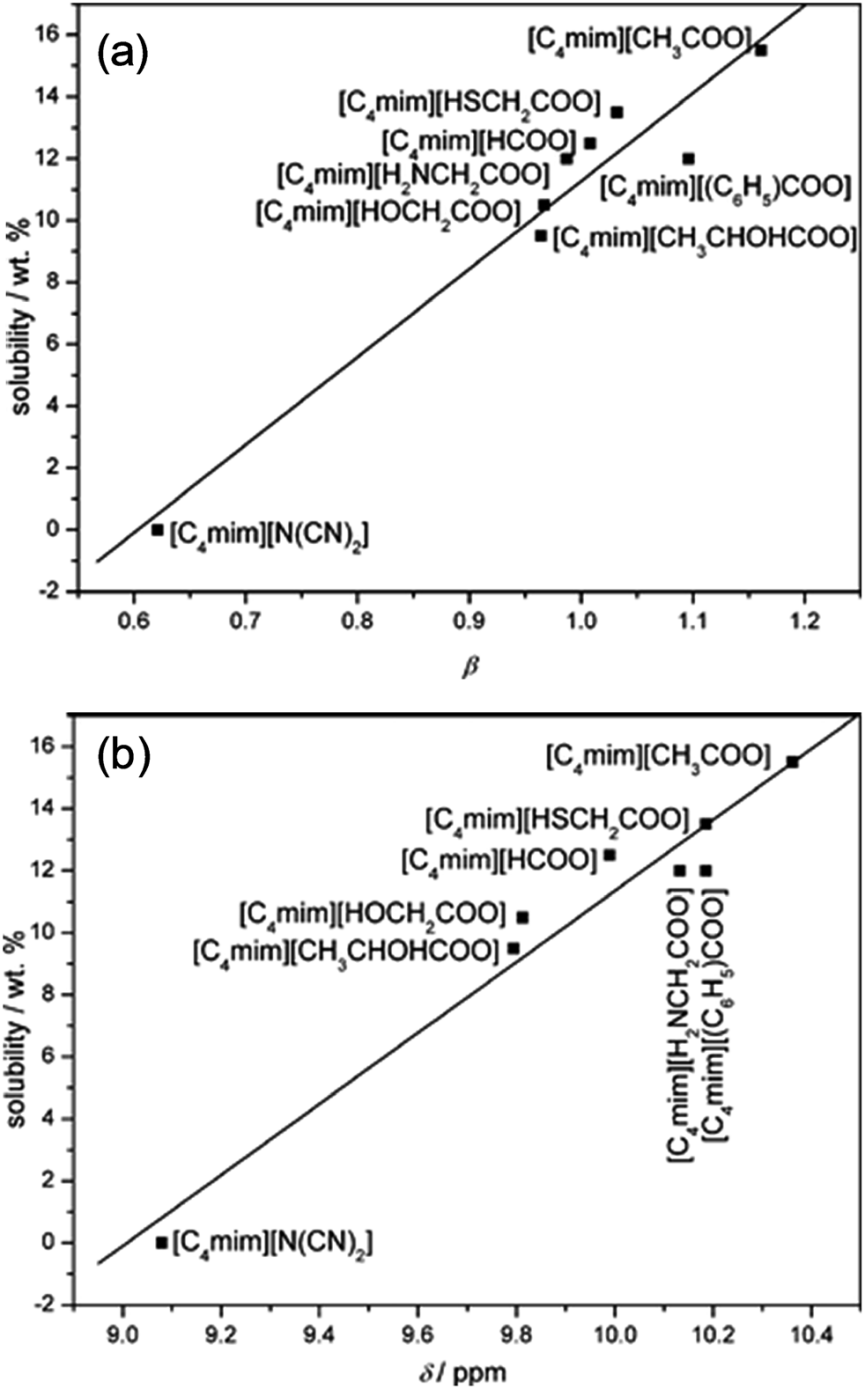
(a) The linear relationship between solubility and H-bond alkalinity. (b) The linear relationship between solubility and the NMR displacement of the imidazole cation C2 position H.^44^ Reproduced from ref. 44 with permission from the Royal Society of Chemistry.

Table 1 summarizes recent experimental studies on the mechanism of cellulose dissolution in ILs. It is known that there are 6 hydroxyl groups in one repeat unit of cellulose, so in the beginning researchers wished to find out what happened between ILs and hydroxyl groups. By using ^13^C and ^35/37^Cl relaxation NMR, Remsing *et al.*^46^,^47^ investigated the interactions of [Emim][Cl], [Bmim][Cl] or [Emim][OAc] with glucose and cellobiose, through which they found that there was no obvious dependence of the relaxation time of cationic carbon nuclei on the concentration of carbohydrate, in contrast to the results of chloride and acetate anions. They also evaluated the ^13^C relaxation time of [Emim][OAc], and found that the reorientation rate of the anion decreased faster than that of the cation. Thus they believed that the interaction of the IL anion and glucose was the governing force, but the cation–carbohydrate interaction was non-specific. Xu *et al.*^44^ examined the relationship between cellulose solubility in ILs and H-bond basicity of anions. They prepared a series of ILs with the same cation [Bmim]^+^ but different Brønsted base anions, and determined the solubilities of cellulose in the ILs and their *β* values. It was found that there was a linear relationship between these two values, as shown in Fig. 3a. The correlation between solubility and the chemical shift of the C2 atom of the imidazolium cation in ^13^C NMR is shown in Fig. 3b. This relationship was also linear because the chemical shift of C2 in the cation was influenced by the H-bond basicity of anions. For other anions like chloride, the linear relationship is not as good as Brønsted base anions.

**Table 1:** Experimental work of mechanistic studies of cellulose dissolution in ILs

Cellulose type	Solvent	Technique	Focus	Reference
MCC	[Bmim]^+^ + anions	^13^C NMR	Anion–cellulose interaction	Xu *et al.*^44^
Carbohydrate	[Bmim][Cl]	^13^C & ^35/37^Cl NMR	Anion–cellulose interaction	Remsing *et al.*^46^
Carbohydrate	[C=C_2_mim]Cl & [Emim][OAc]	^13^C & ^35/37^Cl NMR	IL–cellulose interaction	Remsing *et al.*^47^
Glucose	[Emim][OAc]	Neutron scattering & NMR	IL–cellulose interaction	Youngs *et al.*^48^
Cellobiose	[Emim][OAc]	*In situ* & variable-temperature NMR	IL–cellulose, H-bonds	Zhang *et al.*^49^
Avicel	[Emim][OAc]	WAXS & ^13^C NMR	Anion–cellulose structure change	Endo *et al.*^50^
Avicel	[Emim][OAc]	SAXS & SEM	Cellulose crystallinity	Endo *et al.*^51^
Avicel & α-cellulose	21 ILs	*In situ* microscopy	High-throughput screening	Zavrel *et al.*^52^
Avicel	13 ILs	NMR & Kamlet–Taft parameter	Cation–cellulose interaction	Lu *et al.*^33^
Avicel	9 ILs	^1^H NMR	Odd–even effect of cations	Erdmenger *et al.*^53^
Glucose	[Bmim][OAc]	^13^C NMR	Cation–cellulose reaction	Ebner *et al.*^54^
Avicel, switch grass	[Emim][OAc]	XRD, SANS	Cellulose crystallinity change	Cheng *et al.*^55^
Avicel	[Emim][OAc]/DMSO	XRD, porosimetry	Cellulose crystallinity change	Cheng *et al.*^56^
MCC	[Emim][OAc]/DMSO	Calorimetric method	Heat of dissolution	Andanson *et al.*^57^
Cellobiose	[Bmim][OAc]/[Bmim][Cl]	High-precision solution microcalorimeter	Dissolution enthalpy	Oliveira *et al.*^58^
MCC	[C_2,4,6,8_mim][OAc]/DMSO	Colorimetric method	Alkyl length in the imidazolium cation	Xu *et al.*^59^
MCC	[Bmim][OAc]/DMSO	^13^C NMR	Dissolution mechanism	Xu *et al.*^60^
MCC	[Bmim][OAc]/DMSO	XRD	Co-solvent	Andanson *et al.*^61^
4 cotton stalks	[Emim][OAc]/[Emim]Cl	XRD	Different ILs and particle sizes	Bahcegul *et al.*^62^
5 carbohydrates	11 ILs	HPLC, LCMS, NMR	IL–cellulose reaction	Clough *et al.*^63^
Avicel	7 ILs	*In situ* viscometry measurement	Cellulose dissolution rate	Cruz *et al.*^64^

**Fig. 4:**
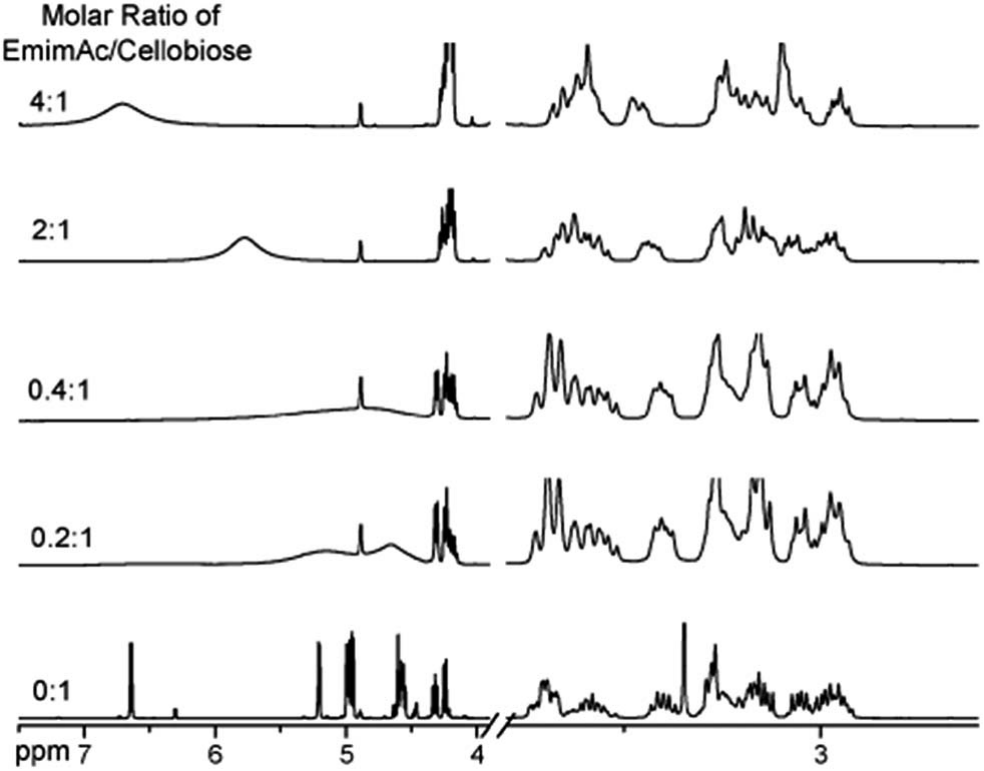
Effects of different ratios of [Emim][OAc]/cellobiose on the nuclear magnetic peak in the ^1^H NMR spectra of cellobiose.^49^ Reproduced from ref. 49 with permission from the PCCP Owner Societies.

Considering the great difference in the mass of different kinds of ionic liquids, Wang *et al.*^32^ converted the unit of the solubility data from gram of cellulose per 100 gram of IL into gram of cellulose per mol of IL (g mol^–1^), and the solubility was found to have a linear correlation with the H-bond basicity of anions, but it is difficult to explain why some ILs containing [Cl]^–^ had much higher solubility. Hardacre *et al.*^48^ arrived at the same conclusion by studying cellulose dissolution in [C_1_mim][Cl] and [Emim][OAc]. Through analysis of the characteristic peak in the ^1^H NMR spectrum of cellobiose in [Emim][OAc], Zhang *et al.*^49^ observed the strong chemical shift of H atoms in disaccharide with increase of the IL concentration, especially for the H in the hydroxyl groups, as shown in Fig. 4. This reflects the formation of a number of H-bonds. Also by *in situ* and temperature-variable ^1^H NMR, they estimated the stoichiometric ratio of [Emim][Ac]/hydroxyl to be between 3 : 4 and 1 : 1 in the primary solvation shell, suggesting that there should be one anion or cation to form H-bonds with two hydroxyl groups simultaneously.

**Fig. 5:**
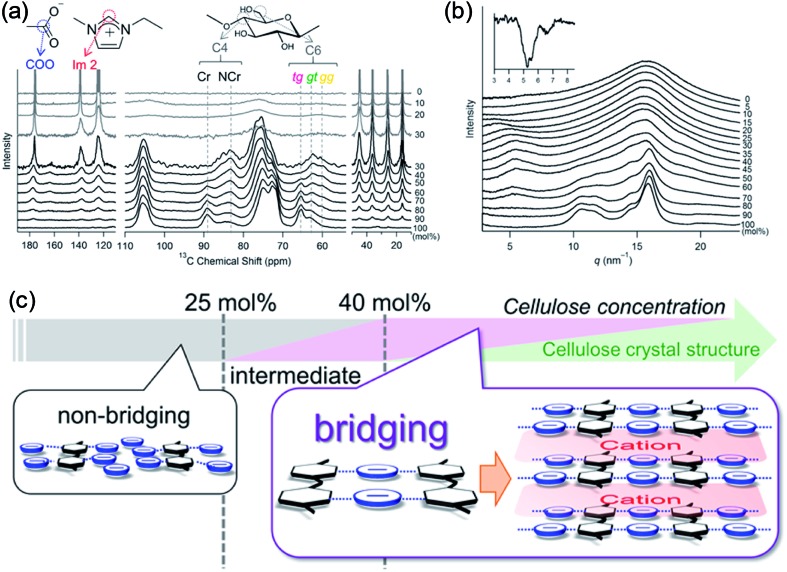
(a) ^13^C solid-state NMR spectra obtained at 5 kHz of MAS for the cellulose/[Emim][OAc] mixtures. (b) WAXS patterns of cellulose/[Emim][OAc] mixtures. (c) Schematic summary of non-bridging to bridging state transformation with the increase of cellulose concentration in [Emim][OAc].^50^ Reproduced from ref. 50 with permission from the American Chemical Society.

Recently, Endo *et al.*^50^ investigated the interactions between cellulose and [Emim][OAc] at different concentrations by wide-angle X-ray scattering (WAXS) and ^13^C solid-state NMR spectroscopy. The results showed that at cellulose concentration of 15–30 mol%, a periodic peak appeared in the WAXS pattern, which corresponded to cellulose chain alignment. At the concentration of >30 mol%, the structure could be changed to ordered layers as [Emim]^+^ and [OAc]^–^ intercalated. They also proposed an anion–bridging structure transformation. At low concentrations of cellulose, the anions interacted with cellulose hydroxyl groups at a 1 : 1 ratio, while at high concentrations, the anions would interact with multiple OH groups resulting in a bridging state, as shown in Fig. 5. Recently, by using small-angle X-ray diffraction (SAXS),^51^ the bridge-like structure of the [OAc]^–^ anion within adjacent cellulose chains linked by OH···O (anion) H-bonds was also suggested, which could accelerate the dissolution process.

Unlike anions, the interaction between cations and cellulose is not strong and cannot be probed easily. According to the viewpoint of Wang *et al.*,^32^ one cannot find an anion that can dissolve cellulose when combined with all kinds of cations, so the cation undoubtedly affects the dissolution in its own way. From the collection of solubility data,^32^ they found that the IL which has a high cellulose solubility would have an anion with strong H-bond basicity and a cation with notable aromatic features, but no quantitative relationship was proposed between the structure and solubility. Zavrel *et al.*^52^ speculated that the aromatic structure of cations could stabilize the H-bonds between the cations and cellulose hydroxyl groups, which needed to be proved by experiments or simulation. Furthermore, through Raman spectroscopy, Ferreira *et al.*^65^ proposed that the imidazolium and pyridinium cations had a much stronger polarization to enhance the dissolution capacity of cellulose in ILs. However, Fernandes *et al.*^66^ did not think there were strong interactions between aromatic cations and anions, so anions would freely interact with cellulose. This was controversial because the remarkable polarization in aromatic cations would result in a strong interaction with anions. Lu *et al.*^33^ used ^13^C NMR to investigate the interaction between cellulose and ILs composed of different kinds of cations and the same acetate anion. Based on the chemical shift, they concluded that there was C–H···O H-bond formation between the cations and cellulose hydroxyl oxygen which influenced the dissolution process, but the chemical shift was not significant and whether the unstable C–H···O could be included as H-bonds should be examined further. In addition, not all the imidazolium cations were effective for cellulose dissolution. Erdmenger *et al.*^53^ proposed an “odd–even” effect, which means that for the ILs with imidazolium cations, only if the number of carbons in the side chain of the imidazolium ring is even the IL can dissolve cellulose efficiently. Marsh *et al.*^67^ suggested that the “odd–even” effect was due to the fact that the carbon number in the side chain had an influence on the polarization of the cations. Besides, there was no more explanation on this “odd–even” effect.

There is also a debate on whether the H-bond can be formed between cations and cellulose. Zhang *et al.*^49^ reported that in their ^13^C NMR results, the chemical shift of cellulose hydroxyl groups was related to the content of ILs, and the broadening of the NMR curve peak corresponding to hydroxyl hydrogen protons resulted from the formation of H-bonds of cations with the hydroxyl groups of cellulose. This suggests that the dissolution was related to both cations and anions. However, this was questioned by Remsing *et al.*^68^ who reanalyzed these NMR results and found that it was not sufficient to arrive at a conclusion on the hydrogenation of cations because the chemical shift was too vague to indicate the impact of cations or other factors. Behind the controversy, it is certain that the cation does affect the dissolution of cellulose in some way. It was also explained by Heinze *et al.*^69^ from carbene formation, who found that the ^13^C NMR signals of certain carbon atoms of the polysaccharide disappeared after dissolving in ILs. They speculated that this was due to the reaction of the reducing end of the polysaccharide with the C2 carbon of the imidazolium cation. From ^13^C isotope labeling and fluorescence labeling experiments, Ebner *et al.*^54^ demonstrated that the C2 atom of [Emim]^+^ could react with the reducing end of the polysaccharide in an alkaline environment. However, the ILs are not an ideal alkaline environment, and the nature of the polysaccharide and realistic cellulose is different, so this interpretation is not universal.

**Fig. 6:**
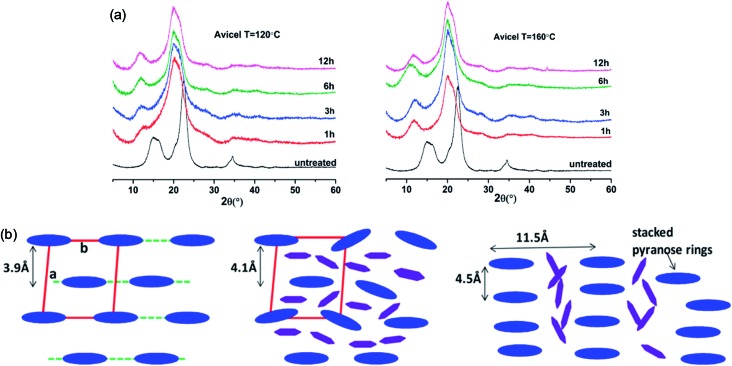
(a) XRD patterns of Avicel cellulose in [Emim][OAc] at 120 °C (left) and 160 °C (right).^55^ (b) Schematic representation of the arrangement of cellulose crystalline structure (blue) and the intercalated ILs (purple).^56^ Reproduced from ref. 55 and 56 with permission from the American Chemical Society.

The change in the crystalline structure of cellulose during dissolution has been reported in several crystallographic studies using X-ray diffraction (XRD) and small-angle neutron scattering (SANS). The native crystalline structure of cellulose is cellulose I and it is also a limiting factor to the utilization of cellulose.^70^ Pretreatment of biomass by ILs typically results in a decrease in cellulose crystallinity as well as transformation of cellulose I to cellulose II, depending on the pretreatment conditions used.^71^–^73^ Cheng *et al.*^55^ found that the Avicel cellulose would suffer a transformation to cellulose II under all processing conditions, as shown in Fig. 6, but for biomass samples, the expansion of cellulose I lattice occurred under all conditions. Temperature, time and the source of cellulose all impacted the change of cellulose crystallinity. They also tried to explain the effectiveness of IL pretreatment by exploring different concentrations of cellulose in [Emim][OAc] solutions.^56^ From XRD measurements, they found the quantitative length change of cellulose I lattice of 3.9 Å to 4.1 Å, which may result from the change of H-bond network inside the crystalline structure. This result showed that the cellulose I lattice expanded and distorted prior to full dissolution in [Emim][OAc], and that the microcrystalline structure led to a less ordered intermediate structure by precipitation, whereas fully dissolved cellulose was like a mixture of cellulose II and amorphous cellulose.

A co-solvent would enhance the interaction between cellulose and ILs to promote the dissolution of cellulose in ILs. Xu *et al.*^59^,^60^ investigated the mechanism of dimethyl sulphoxide (DMSO) as a co-solvent to increase the solubility of cellulose in ILs. They analyzed the chemical shift of the cations in different concentrations of DMSO/[Bmim][OAc] by ^13^C NMR. It was found that DMSO changed the solvated structure around the aromatic cations, which may be the reason for the solubilization effect of the DMSO co-solvent. In recent years, a series of reports have indicated that ionic liquids are not non-derivatized solvents for cellulose. Clough *et al.*^74^ found that with the extension of dissolution time of cellulose in [Emim][OAc], more complex chemical shifts were observed in the ^1^H NMR spectrum, indicating the reaction product formation of the cation with the reducing acetal end of the glucose unit. They suggested that altering the structure of the [OAc]^–^ anion could make it difficult for the imidazolium to form a carbene structure, thereby maintaining the stability of the cellulose structure in ILs without much change in solubility.

## 4. Computational studies on why ILs dissolve cellulose

**Fig. 7:**
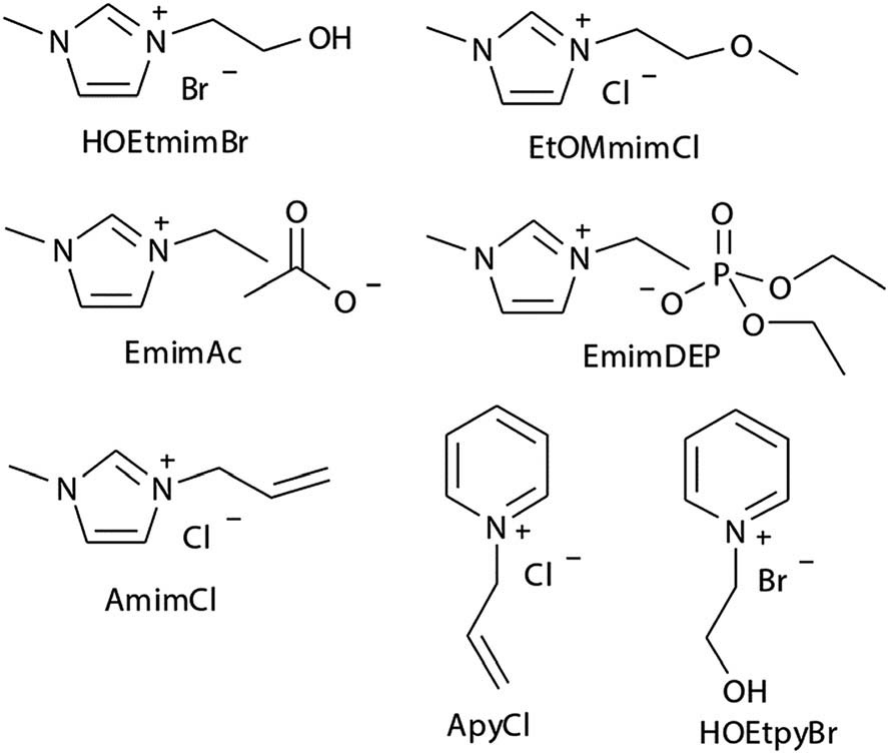
The selected 7 ILs predicted by Liu *et al.* using COSMO-RS.^78^ Reproduced from ref. 78 with permission from the Royal Society of Chemistry.

Experimental studies can provide reasonable conclusions for the interactions of cellulose with ILs, but still lack molecular level understanding. Computational methods, such as COSMO-RS, quantum chemistry calculation and molecular dynamics simulation, can reveal structural details at the atomic level, thus plenty of inspiring results have been reported for the dissolution mechanism based on these techniques. We have summarized recent computational studies on cellulose dissolution in ILs, as tabulated in Table 2. In the early stage, several studies of glucose and cellulose oligomers with ILs were performed using the conductor like screening model for real solvents (COSMO-RS).^75^–^78^ Kahlen *et al.*^75^ used COSMO-RS to screen more than 2000 kinds of ILs, and studied their interactions with trisaccharide. It was found that anions of the ILs easily accepted H-bonds, so the anions dominated the dissolution process. Casas *et al.*^76^ studied the solubility of cellulose and lignin in 780 ILs by using the calculation method of activity coefficient and excess enthalpy in COSMO-RS. They demonstrated that when cellulose and lignin in ILs had low activity coefficient and the dissolution process was exothermic, ILs always showed high solubility for cellulose. After that, they expanded the system to a 3 × 3 cellulose monolayer model and calculated the optimal configuration, the activity coefficient and excess enthalpy after mixing with each of the 12 ionic liquids, and the results were still consistent with the experiments. Recently, Liu *et al.*^78^ used COSMO-RS to predict ILs with novel structures for cellulose dissolution, which was the first work to guide the design and synthesis of new IL solvents. They established a model using COSMO-RS to predict the solubility of cellulose, and the model was applied to the screening of new kinds of ILs. Then, 7 kinds of ILs with good solubility were chosen and synthesized as shown in Fig. 7. The solubility of cellulose in these ILs measured by experiments was consistent with that predicted by the model mentioned above. By calculating the excess enthalpy, they found that the H-bonds between anions and cellulose were determinant in the dissolution process, and the design principles of the anions, cations and the types of cationic substituents were refined by the data from the prediction model.

**Table 2:** Simulation work of mechanistic studies of cellulose dissolution in ILs

Cellulose model	Solvent	Method/force field/basis set	Focus	Reference
Cellotriose	>2000 ILs	COSMO-RS	Solubility modeling	Kahlen *et al.*^75^
Glucose	320 ILs	COSMO-RS	Solubility prediction	Casas *et al.*^76^
3*3 structure	750 ILs	COSMO-RS	Cellulose solubility & activity coefficients	Casas *et al.*^77^
1,3,4-mer oligomers	357 ILs	COSMO-RS	Solubility prediction	Liu *et al.*^78^
Cellobiose	[Bmim][Cl]	DFT [6-31G(d)]	IL–cellulose interaction	Novoselov *et al.*^79^
Dimethoxy-glucose	[Emim][OAc]	DFT [6-31+G(d)]	IL–cellulose interaction	Ding *et al.*^80^
Glucose	[C_1_mim][Cl]	DFT-D, 6-311++G(2d,2p)	IL–cellulose interaction	Janesko *et al.*^81^
Cellobiose	[Bmim][Cl]	DFT [6-311+G(d,p)]	IL–cellulose reaction	Yao *et al.*^82^
2,4,6-mer oligomers	[Bmim][Cl]	DFT/MD, Glycam/6-311+G(d,p)	Dissolution mechanism	Xu *et al.*^83^
10-mer oligomer	[Bmim][OAc]	DFT/MD, Glycam/6-311++G(d,p)	Co-solvent	Zhao *et al.*^84^
Glucose derivatives	[C_*n*_mim][Cl]	MD [compass]	Derivative compatibility	Derecskei *et al.*^85^
Glucose	[C_1_mim][Cl]	MD [OPLS-AA]	IL–cellulose interaction	Youngs *et al.*^86^
Glucose	[Emim][OAc]	MD [OPLS-AA]	IL–cellulose interaction	Youngs *et al.*^48^
Glucose	[Emim][OAc]	MD [OPLS-AA]	Co-solvent	Andanson *et al.*^61^
Glucose	[Bmim][Cl]	MD [Glycam]	Thermodynamic research	Jarin *et al.*^87^
Glucose & cellobiose	[Emim][OAc]	MD [Glycam]	Thermodynamic research	Bharadwaj *et al.*^88^
Cellobiose	[Bmim][Cl]	MD [OPLS]	IL–cellulose reaction	Li *et al.*^89^
5,10,20-mer oligomers	[Emim][OAc]	MD [Glycam]	IL–cellulose interaction	Liu *et al.*^90^
10-mer oligomer	[C_*n*_mim][Cl]	MD [Glycam]	Cationic structure	Zhao *et al.*^91^
10-mer oligomer	[Emim]^+^ + anions	MD [Glycam]	Anionic structure	Zhao *et al.*^92^
10-mer oligomer	[Bmim][Cl]	MD [Glycam]	Thermodynamic research	Mostofian *et al.*^93^
I_β_ crystal	[Bmim][OAc]	MD [AMBER]	Dissolution mechanism	Gupta *et al.*^94^
I_β_ crystal	[C_*n*_mim][Cl]	MD [OPLS-AA]	Co-solvent	Huo *et al.*^95^
Microfibril	[Bmim][Cl]	MD [Glycam]	Dissolution mechanism	Mostofian *et al.*^96^,^97^
Microfibril	[Emim][OAc]	MD [Glycam]	Cellulose conformation	Liu *et al.*^98^
Microfibril	[Bmim][Cl]	MD [Charmm]	Thermodynamic research	Chu *et al.*^99^–^101^
Cellulose bunch	[Bmim][Cl]	MD [OPLS]	H-bonds & dissolution mechanism	Ismail *et al.*^102^–^104^
Cellulose bunch	[Emim][OAc]	MD [Glycam]	Dissolution mechanism	Li *et al.*^105^

Another widely used method to study the interactions of cellulose with ILs is quantum chemistry calculation (QM). Ding *et al.*^80^ used DFT calculation to study the interaction between [Emim][OAc] and glucose, and they found that the H-bond formed between IL and cellulose was stronger than that formed inside the cellulose molecules, which resulted in the dissolution of cellulose in ionic liquids. And the potential energy of glucose with anions was higher than that of glucose with cations, indicating that the anions played a major role in the dissolution process and the role of cations was secondary. Payal *et al.*^106^ used cellobiose as a model compound to study its interaction with multiple ILs. It was found that the stability of the disaccharide conformation was dependent on the intramolecular H-bonds, and the H-bonds between the anion and hydroxyl groups could replace the original H-bonds in cellulose. According to their results, both anion and cation played a role in the process. Yao *et al.*^82^ selected cellobiose as a model compound, and [Bmim][Cl], [Emim][Cl] and [Emim][OAc] as the solvents to study their interactions. It was found that the IL cation could react with the cellulose reducing terminal, and the cation and anion both played a catalytic role in promoting the dissolution of cellulose. Anions such as [Cl]^–^ could reduce the potential barrier of catalytic cracking of cellobiose.

**Fig. 8:**
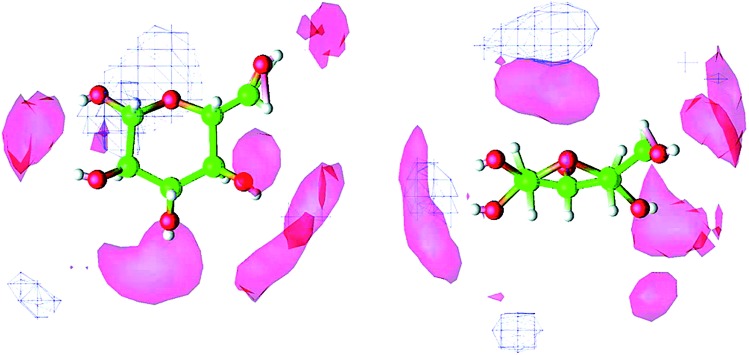
The spatial distribution function of [Bmim][Cl] around glucose. The left is the top view, the right is the side view, the anion is in the red area and the cation is in the white grid area.^107^ Reproduced from ref. 107 with permission from the American Chemical Society.

Molecular dynamics (MD) simulation is the most commonly used method in the mechanism study. The current research approach can be classified into two types. One is the direct unbiased molecular dynamics simulation used to study the dissolution process, and the other is through complex and advanced sampling methods in more diverse phase space to deal with the system to obtain the physical quantity of interest. Derecskei *et al.*^85^ carried out the first molecular dynamics simulation for the interactions of ILs with cellulose. The interactions of different lengths of polysaccharide polymers with IL were studied. It was found that the solubility was linearly related to the degree of polymerization (DP) of cellulose. Youngs *et al.*^86^ used a single glucose to represent cellulose, then simulated it with 128 pairs of [Bmim][Cl]. They found that [Cl]^–^ could form a stable H-bond with the hydroxyl groups of glucose, and cations also had a weak interaction with glucose.^107^ From the spatial distribution function (SDF) shown in Fig. 8, the solvated structure of glucose in [Bmim][Cl] could be clearly understood. [Cl]^–^ was distributed in the first solvation shell, and about 3–4 [Cl]^–^ anions interacted with one glucose to form hydrogen bonds, which was consistent with the NMR results of Remsing *et al.*^46^ In the second solvation shell, the imidazolium cation was mainly distributed in the upper and lower positions of glucose. The calculated energy also indicated that the hydrogen bond between the anion and the cellulose was the main source of the interaction energy.

**Fig. 9:**
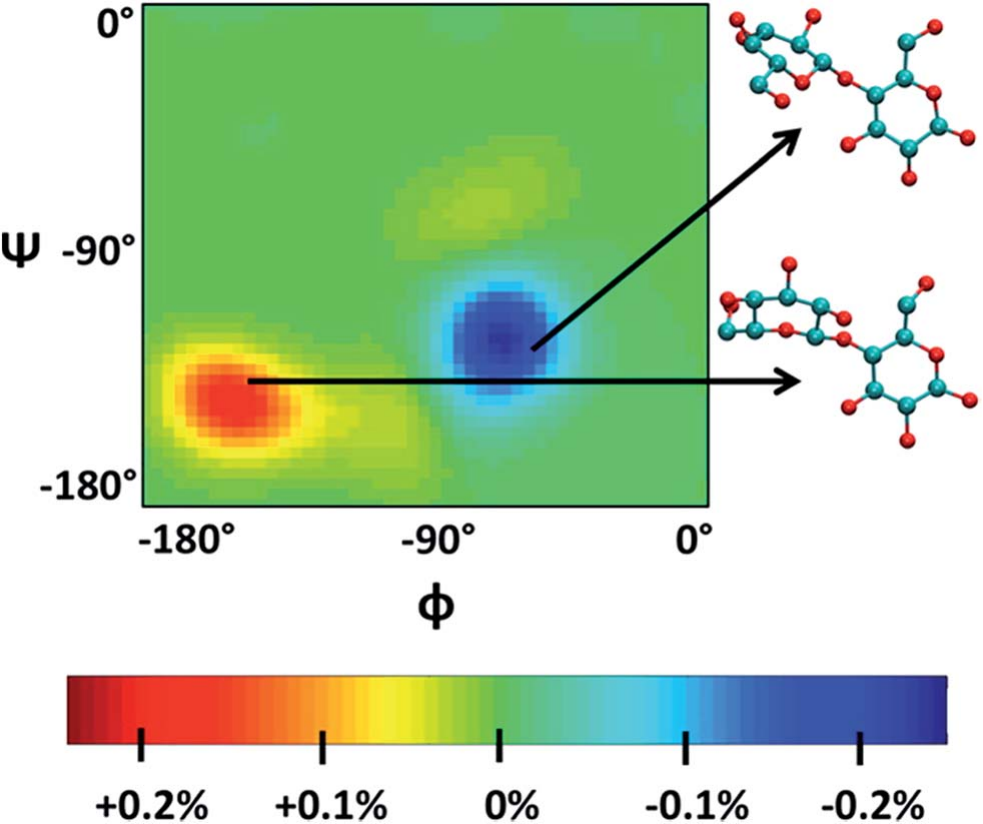
*φ*–*ψ* difference maps ([Bmim][Cl]–water) for the most populated basin O (–180° < *φ*/*ψ* < 0°) at 375 K and 450 K. The two examples of cellobiose structures characterize the difference between structures from the “[Bmim][Cl] basin” (red) and those from the “water basin” (blue).^93^ Reproduced from ref. 93 with permission from the American Chemical Society.

Singh *et al.*^90^ put a DP = 8 cellulose chain into [Emim][OAc] for molecular dynamics simulation. It was shown that acetate anions formed multiple H-bonds with one cellulose chain, while some cations were in contact with polysaccharide chains through hydrophobic interaction. Compared with water, the polysaccharide in the IL tended to have a larger stretching angle for the 1–4 glycosidic bond, leading to the crystallinity change of cellulose before and after dissolution. Zhao *et al.*^91^ systematically studied the influence of different types of cations and anions on the dissolution. By comparing the radial distribution function (RDF) and the interaction energies, it was found that the shorter the alkyl chain of the cation, the stronger the interaction of the ILs with cellulose. The side chain in the imidazolium cation with electron donating groups promoted the dissolution of cellulose; for example, with an unsaturated side chain structure, [Amim]^+^ cation was more likely to interact with cellulose. However, the electron withdrawing groups would hinder the interactions of the ILs with cellulose. After that, they studied the anion effect,^92^ and found a decreased order of interaction energy as [Cl]^–^ > [Ac]^–^ > [(CH_3_O)PO_2_]^–^ > [SCN]^–^ > [PF_6_]^–^ when combined with the [Emim]^+^ cation. They also proposed a strategy for screening anions, that is, the anions must have a higher electron density, shorter side chains, and with no electron withdrawing group. In recent years, there have been reports with attempts to find new IL solvents by molecular dynamics simulations. Timothy *et al.*^108^ simulated the mixture of [Me(OEt)_3_EtIm][OAc] with glucose or cellobiose, and found that the IL was able to maintain strong anionic basicity while the cation was more likely to change the spatial configuration of glucose, so that the interaction of cellulose with anions was stronger. Marta *et al.*^109^ studied the interactions between glucose and several cyano-based ILs. They found that besides strong electrostatic attraction with glucose, the viscosity of the ILs was very low, showing industrial application prospects. In order to explore the interactions between cellulose and ILs in more detail, Mostofian *et al.* studied the interactions of a single DP = 10 cellulose chain with [Bmim][Cl] and water using replica-exchange molecular dynamics (REMD), focusing on the conformation change of the cellulose chain.^93^ As shown in Fig. 9, they found that the cellulose chains in the IL were more prone to bending, where the H-bonds inside the single chain were destroyed. The study of the conformation entropy indicated that the degree of freedom of a single cellulose chain in the ILs was greater, suggesting that the dispersion of cellulose chains in ILs was more likely to occur.

**Fig. 10:**
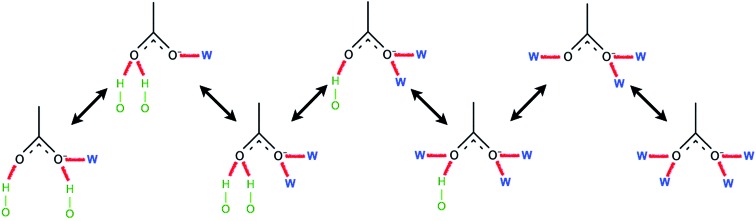
Typical path of a free acetate anion to and from one state when water is present.^104^ Reproduced from ref. 104 with permission from the PCCP Owner Societies.

Ismail *et al.* carried out a series of MD simulations for the interaction of [C_*n*_mim][Cl] with a cellulose chain.^103^ The cellulose was divided into polar and non-polar regions, where the hydroxyl group was the polar region and the glucan ring was the non-polar region. It was shown that the anion mainly interacted with the polar region by electrostatic attraction, and the cation interacted with the non-polar region by the van der Waals force to form an patchwork interaction model. In addition, they also analysed the H-bond conformation migration of different anions in different systems.^104^ The results showed that the H-bonds formed by [OAc]^–^, [Cl]^–^ and [DMP]^–^ could not be easily replaced by the internal H-bonds of cellulose. The addition of water broke the stability of the H-bond conformation. Fig. 10 is the migration path of the H-bond conformation of acetate and cellulose in the presence of a large amount of water.

## 5. Cellulose microfibril dissolution and the synergistic mechanism

**Fig. 11:**
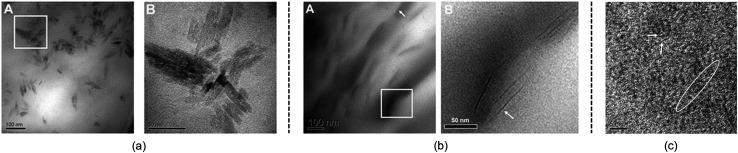
TEM photographs of microcrystalline cellulose at different scales treated by [Amim][Cl] after 1 h. (a) is 100 nm, (b) is 50 nm, (c) is 5 nm (room temperature for 4 months).^111^ Reproduced from ref. 111 with permission from the Royal Society of Chemistry.

Although the studies mentioned above provide useful knowledge of the dissolution mechanism, there are still some problems. One of the most notable problems is that using glucose, cellobiose and polysaccharides as model compounds for cellulose is not appropriate. In fact, a polysaccharide with DP < 6 can dissolve in water,^110^ which is much different from realistic cellulose. As stated in the Introduction, it is the crystalline form of natural cellulose that results in the stability of cellulose in common solvents.^8^,^16^ Therefore, a lot of scientists have made efforts to study a larger cellulose microfibril which may be more realistic. Zhang *et al.*^111^ used transmission electron microscopy (TEM) to study the dissolution of microcrystalline cellulose in [Amim][Cl]. It was found that the structure of the filament was destroyed in the initial step, and then cellulose filaments dispersed, followed by the gradual dissolution of the cellulose, and finally the system was stabilized in a mixture of irregular cellulose chains and IL, as shown in TEM pictures in Fig. 11.

**Fig. 12:**
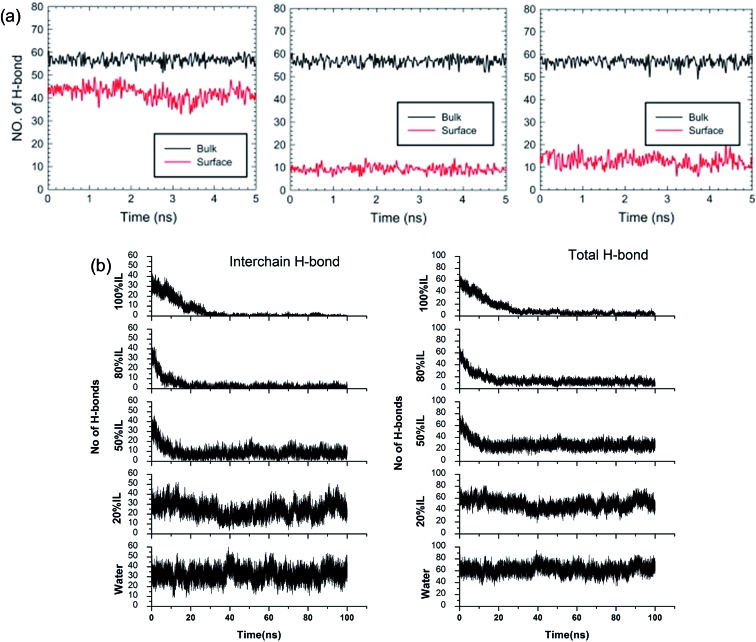
(a) The change of H-bonds inside the cellulose layer (black) and at the surface (red). From left to right is [Bmim][PF_6_], [Bmim][OAc] and H_2_O.^94^ (b) Effect of [C_2_mim][OAc]–water mixtures on the disruption of the inter-chain H-bonds and total H-bonds between cellulose at 160 °C.^42^ Reproduced from ref. 42 and 94 with permission from Elsevier and the Royal Society of Chemistry.

Although the TEM experiments give us some new insights into the dissolution process, atomistic details of the cellulose–IL system are still indirect, for which MD simulation has a particular advantage. Gupta *et al.*^94^ put the cellulose microfibril model of cellulose I_β_ chains at the bottom of a simulation box and the remainder space filled with the solvent to study the interactions of [Bmim][OAc], [Bmim][PF_6_] and H_2_O with the cellulose surface. It was found that there were many H-bonds formed between adjacent cellulose chains. As shown in Fig. 12a, when the cellulose was in contact with water and [Bmim][PF_6_], the number of H-bonds did not decrease, but when in contact with [Bmim][OAc], the number decreased significantly. This result showed that the interchain H-bond broken by ILs is the main factor in the dissolution of cellulose. It was also shown that a trace amount of water did not affect cellulose dissolution in ILs. By simulating a cellulose I_β_ bundle in [Emim][OAc]/water mixtures, Shi *et al.*^42^ found that small amounts of water only had a small effect on the H-bond disrupting ability of the IL, and the reduction in the number of H-bonds was directly proportional to the concentration of [Emim][OAc], as shown in Fig. 12b.

Ismail *et al.*^102^ established a cellulose bundle with six single chains of DP = 8 in [Bmim][Cl], [Emim][OAc] and [C_1_mim][DMP]. The structure of the bundle changed a lot after putting in the ILs, especially in [Emim][OAc], where the cellulose bundle showed a preliminary dispersion trend. The anions were found to tightly bind around the hydroxyl groups of the cellulose bundles and formed a negatively charged complex, to which the cation was also close due to electrostatic attraction. They believed that the cations entered into the cellulose chains and played an important role in dissolution. Considering the fact that cellulose microfibril in the cell wall of higher plants is composed of 36 long-polymerized cellulose chains, Mostofian *et al.*^96^ conducted all-atom MD simulations of a 36-chain cellulose microfibril in [Bmim][Cl] and water for 100 ns. It was found that [Cl]^–^ interacted with hydroxyl groups in different cellulose layers and [Bmim]^+^ stacked preferentially on the hydrophobic cellulose surface, stabilizing detached cellulose chains.

**Fig. 13:**
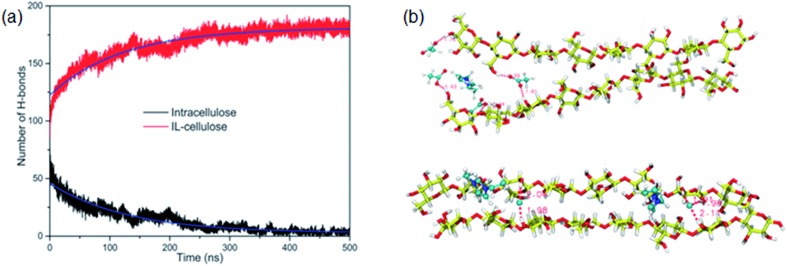
(a) The change of intracellulose and cellulose–IL H-bonds during the simulation of a cellulose bunch in [Emim][OAc]. (b) Snapshots of H-bond formation between cellulose chains and ILs.^105^ Reproduced from ref. 105 with permission from the PCCP Owner Societies.

The whole dissolution process of cellulose from the crystal structure to dispersed cellulose chains was first revealed by us.^105^ Through 500 ns MD simulation, a cellulose bunch consisting of 7 glucan chains (DP = 8) was put in [Emim][OAc] and [Emim][Cl] to investigate the dissolution process. We found that complete dissolution happened in [Emim][OAc], with every single chain separated from each other. As shown in Fig. 13a, the number of intracellulose H-bonds and cellulose–IL H-bonds became constant after 350 ns in the MD simulation, which means that the original H-bond network was destroyed by ILs and replaced by a new anion–cellulose H-bond network. Moreover, [OAc]^–^ could form three different kinds of H-bonds within cellulose chains which provided sufficient gaps for separation. However, [Cl]^–^ could not effectively divide the cellulose chains, as shown in Fig. 13b, and this is why [OAc]^–^ is more effective in the dissolution of cellulose. In addition, a synergistic mechanism of cation and anion in the dissolution of cellulose was investigated. It was shown that the anions initially formed H-bonds with the hydroxyl groups of cellulose by insertion into the cellulose strands, and cations stacked to the side face of the glucose rings. As more and more anions bound to the cellulose chains, cations started to intercalate into the cellulose bunch due to their strong electrostatic interaction with anions and van der Waals interaction with the cellulose bunch. Then cellulose dissolution begins. To study the role of cations in the dissolution process, we further investigated the controlling mechanism of the unsaturated structure of cations in the dissolution of cellulose in ILs.^27^ The changing process of the cellulose bunch in [Bmim][OAc], [Bpyr][OAc], [Bpy][OAc] and [Bpip][OAc] was simulated, respectively. It was found that cellulose could only be dissolved in the ILs containing unsaturated cations. The influence of the heterocyclic ring was analyzed by quantum chemistry calculation and the effect on the mass transfer was also examined. The unsaturated heterocyclic structure was found to affect the dissolution from two aspects. One is the structure factor: the π electron delocalization of the unsaturated heterocyclic ring makes the cation more active to interact with cellulose and provides more space for acetate anions to form hydrogen bonds (H-bonds) with cellulose. The other is the dynamic effect: the larger volume of cations with saturated heterocyclic rings results in a slow transfer of both cations and anions, which is not beneficial to the dissolution of cellulose.

**Fig. 14:**
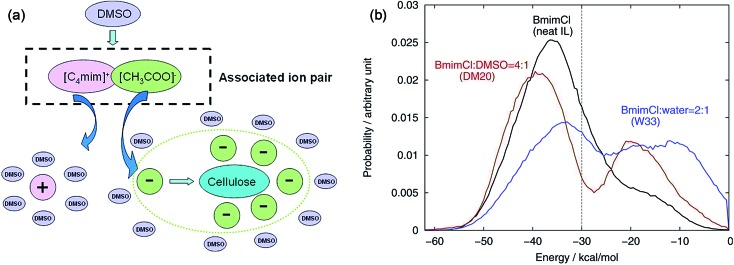
(a) Co-solvent effect of DMSO.^84^ (b) PED energy variation of typical solvent systems.^95^ Reproduced from ref. 84 and 95 with permission from the American Chemical Society.

The mechanism of the role of co-solvent in promoting the dissolution of cellulose in ILs was also investigated by MD simulation extensively. Zhao *et al.*^84^ investigated the effects of adding DMSO, DMF, CH_3_OH and water on cellulose dissolution in [Bmim][OAc] *via* MD simulation and quantitative calculation. They presented the mechanism of enhancement of cellulose dissolution by the co-solvent as shown in Fig. 14a. When adding a certain percentage of aprotic solvents such as DMSO into an IL, a solvation layer around the cation was formed by the molecular solvent, weakening the interaction between the cation and anion. On the other hand, the aprotic solvent also had a solvation effect on the aggregates formed by cellulose and anions to stabilize the dissolved system, which promoted the dissolution of cellulose. In contrast, the protic solvent could strongly solvate the acetate anion, hindering the formation of H-bonds between the anion and cellulose. At the same time, Huo *et al.*^95^ proposed an indicator, named PEDs (pair energy distributions), to evaluate the interaction energy between the solvent and the surface units of glucose. As shown in Fig. 14b, when the PED was greater than 30 kcal mol^–1^, the solvent was able to dissolve cellulose, but it could not dissolve cellulose at the PED value of 30 kcal mol^–1^ or less. The PED indicator may offer a reference for the design of new solvents and co-solvents. In the above two research studies, the co-solvent was regarded to have a structural effect on IL–cellulose interaction, while some workers believed that the co-solvent effect resulted from the improved mass transport of ILs. Recently, Sadiye *et al.*^112^ investigated a cellulose I_β_ model in a mixed solvent of IL and DMSO, and they suggested that DMSO improved the mass transfer in the system and promoted the interaction between ions and cellulose, but would not change the structure of cellulose and cellulose bundles, which may inspire the future design of new cellulose solvents. Through MD simulation, Ramakrishnan *et al.*^113^ found that when water mass fraction did not exceed 40%, [Emim][OAc] aqueous solution could dissolve cellulose, and the dissolution became faster because the addition of water greatly reduced the solvent viscosity.

With all the above discussion, we can come to a general understanding of the process of cellulose dissolution. Firstly, the cations and anions come into contact with the surface of cellulose microfibril, and the H-bond formed by anions changes the orientation of cellulose hydroxyl groups, making the surface chains swelling to the solution. Through the concerted action of cations and anions, the cellulose chains are gradually peeled from the crystal and cellulose forms less uniform aggregates with ions until fully dissolved in and surrounded by ILs. However, in the synergistic mechanism, anions play a more important role than cations. As the synergistic mechanism is mainly arrived at from simulation results, more experimental evidence is still needed to make it more convincible.

## 6. Physicochemical aspects of cellulose dissolution in ILs

**Fig. 15:**
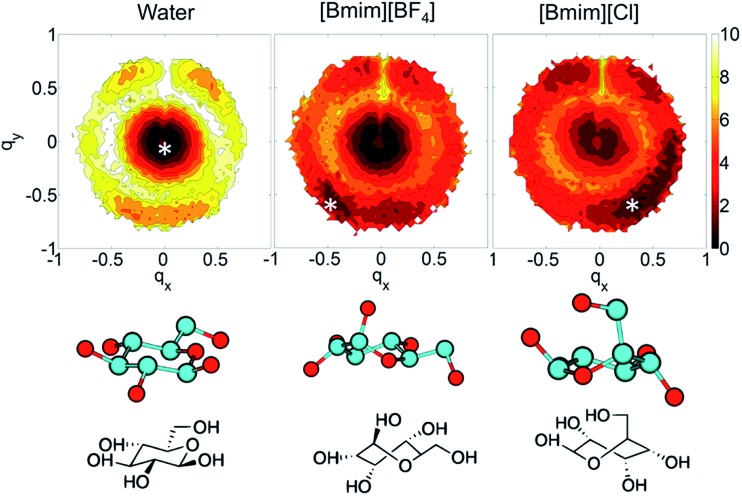
The free energy distribution of the glucose ring conformation in the three solvents. *q*_*x*,*y*_ is the wrinkle coordinate, and the color depth represents the probability density. The bottom part below the snowflake symbol is the glucose configuration.^87^ Reproduced from ref. 87 with permission from the American Chemical Society.

In the investigations of cellulose dissolution in ILs, researchers used calorimetric methods to determine the enthalpy change of microcrystalline cellulose dissolving in imidazolium ILs.^57^,^58^ A recent research used microcalorimetry to accurately determine the enthalpy change of microcrystalline cellulose dissolution in [Emim][OAc].^57^ It was shown that the dissolution of 1 g of cellulose in [Emim][OAc] released –132 ± 8 J heat, and because the degree of freedom of the system does not change much, it can be inferred that this is a thermodynamically permissible exothermic process. The mixing heat was also found to be a good indicator of cellulose dissolution in ILs. Oliveira *et al.*^58^ arrived at the same conclusion through a similar calorimetric method. However, the experimental studies are very limited and determination of the heat value of realistic fibril is not as easy as glucose or cellobiose. A statistical thermodynamic method has also been used to calculate the free energy in the dissolution process. Recently, Rajdeep *et al.*^114^ studied the change of dissolution free energy of cellobiose in ILs with different anions. It was found that the dissolution process was enthalpy-driven and closely related to the H-bonds formed by cellulose in the system. The free energy results also showed that [OAc]^–^ was an effective anion for the ILs. Jarin *et al.*^87^ used a PTMetaD–WTE approach to study the equilibrium glucose ring structure in [Bmim][Cl] and [Bmim][BF_4_], and new insights into potential energy surface were provided towards the dissolution mechanism. They calculated the exact change of the conformational free energy of glucose in an explicit solvent environment, and found that the ring structure of glucose was significantly different in different solvents. As shown in Fig. 15, the glucose rings were more distributed in the low conformation free energy area in [Bmim][Cl]. Vivek *et al.*^88^ used a similar method to study the behaviour of glucose and cellulose disaccharides in imidazolium-like ILs with different side chain lengths. The results showed that the dihedral angles of glucose C6 position and the linked glucosidic bond in disaccharide had low rotational free energy in ILs, which may be the reason for the change in cellulose crystallinity or even degradation in ILs.

**Fig. 16:**
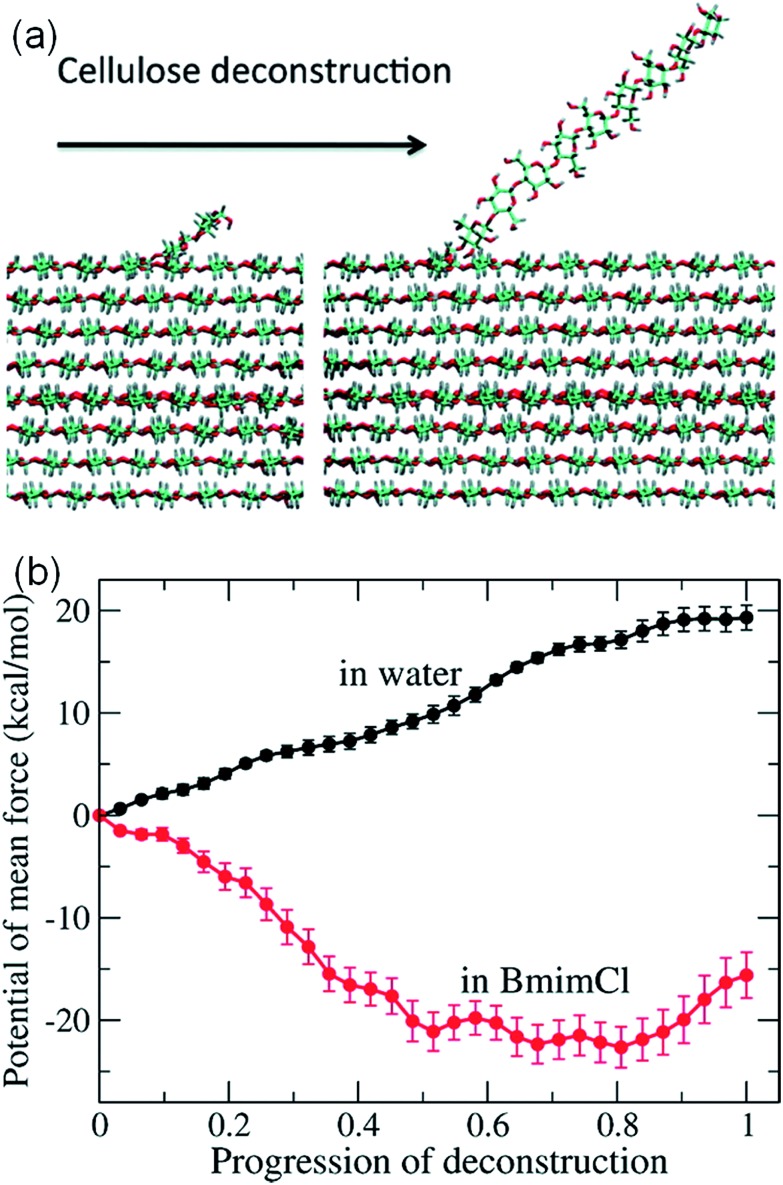
(a) The peeling process of cellulose by means of steered molecular simulation. (b) The potential mean force (PMF) of the peeling process in water and ionic liquids.^99^ Reproduced from ref. 99 with permission from the American Chemical Society.

It is challenging to simulate the free energy change of a realistic cellulose microfibril during the dissolution process. Chu *et al.*^101^ used a biased sampling method to explore the decisive factors of cellulose dissolution in ILs. By considering two extreme dissolution states in IL and water of a cellulose microfibril composed of 36 single chains, they discussed the effect of solute structure on the entropy increase of the system, and proposed two dissolution determinants at the molecular level. One is the subtle impact of dissolved cellulose chain on the solvent structure, and the other is the interaction of cellulose microfibril layers weakened by the cation and anion. They believed that an effective solvent for cellulose dissolution should have the following characteristics: (i) the solvent should interact with cellulose fibril in the axial direction, and (ii) it should break the interaction between cellulose layers. Later, the contribution of entropy increase in the dissolution process of cellulose in IL and water was studied through MD simulation using a biphasic thermodynamic model.^100^ In water and [Bmim][Cl], the dispersion of cellulose led to a change in the degree of freedom of the solvent and a reduction in entropy, but the magnitude of entropy reduction in [Bmim][Cl] was much smaller. Considering from the viewpoint of entropy and the interaction, [Bmim][Cl] was more suitable for cellulose than water. They also calculated the free energy change of the peeling-off process of cellulose microfibril in water and [Bmim][Cl].^99^ For this purpose, the steered molecular dynamics method was used to peel off a cellulose chain from the surface of a cellulose microfibril (Fig. 16a). Through the reaction coordinates provided by the peeling process, the authors used their in-house code to calculate Gibbs free energy during the peeling process, as shown in Fig. 16b. The peeling of the cellulose chain in [Bmim][Cl] was found to be a process of free energy reduction, with about 2 kcal per mol per glucose, but the same process in water is a free energy increasing process. They also set up a set of coarse-grained force field for the IL to calculate the interactions of glucose with the IL. The result indicated that the anion interacted with the hydroxyl groups of glucose, and the cation was more likely to interact with the glucan chain and the ether oxygen.

Generally speaking, the cellulose dissolution in “good” IL solvents is a Δ*G* < 0 process, *i.e.*, it is a spontaneous exothermic process. Combined with the experimental and simulation studies in previous sections, the structural features of cellulose hydroxyl groups make it easy to form strong H-bonds with anions of ILs, even much stronger than the original H-bond network inside the cellulose structure. This would be the inherent nature for the cellulose dissolution at the molecular level. An effective IL for dissolving cellulose should have suitable cationic and anionic structures, to interact with cellulose strongly and make it more favourable to dissolve in the IL.

Temperature is one of the most important factors that affect the dissolution of cellulose mainly through the transport properties. Fukaya *et al.*^115^ reported that increasing the temperature could reduce the dissolution time and increase the solubility. Considering the fact that the melting point of chloride ILs is always higher and difficult to use, they developed phosphate salt ILs to dissolve cellulose at 45–65 °C. In the investigation of cellulose dissolution in ILs, the temperature is generally above 80 °C, at which the viscosity is 147 mPa s for [Bmim][Cl], 62 mPa s for [Emim][Cl], 10 mPa s for [Emim][OAc], much smaller than the values at room temperature. However, the necessity of employing higher temperature results in high energy consumption, which is not desired for green and sustainable processing. Andanson *et al.*^61^ found that complete dissolution of cellulose at a lower temperature could be achieved by reducing the heating rate. They performed a series of experiments at different operating temperatures and different heating rates, through which they observed that for the 5 wt% cellulose/IL system, the heating rate of 1 °C min^–1^, 0.1 °C min^–1^ and 0.01 °C min^–1^ could decrease the complete dissolution temperature from 72 °C and 55 °C to 34 °C, respectively, which made the application at lower temperature possible. They presumed the reason to be the effect of mass transfer on the dissolution process.

Viscosity affects the speed of dissolution and is the most important kinetic factor, which has much impact on the transport of ILs, the contact efficiency between ILs and cellulose, and the better application potential in the subsequent recovery step. Usually, ILs containing long alkyl chains demonstrate higher viscosity, and the cations with short alkyl chains and unsaturated groups show better transport properties.^27^,^52^ As a kind of ILs with an allyl group in the cation, [Amim][Cl] has much lower viscosity, faster dissolution kinetics, and higher dissolution efficiency. Cruz *et al.* used *in situ* viscosity measurements to study the rate of cellulose dissolution in a number of ILs,^64^ and [Bmim][OAc] was found to dissolve cellulose faster than analogous ILs and the rate of dissolution was affected by both anion basicity and its relative concentration. Fukaya *et al.*^115^ stated that the solubility decreased with the increase of viscosity, and the bigger size of cations led to higher viscosity. However, the viscosity of [Emim][Cl] is smaller but cellulose solubility is not as high as [Bmim][Cl], and the formate ILs have much lower viscosity, and even lower solubility than acetate ILs, thus viscosity is not a precise indicator. Some researchers^57^ demonstrated that viscosity was significant in controlling the dissolution of cellulose in ILs. They thought that the dissolution of cellulose in ILs itself was a thermodynamically acceptable process, and the difference in solubility resulted from the change in viscosity. Changing the temperature and adding a co-solvent could improve the viscosity of the system, so that the solubility increased. However, from the viewpoint of chemical thermodynamics, solubility is an equilibrium thermodynamic property, and low viscosity can only shorten the time of achieving dissolution equilibrium, but cannot affect the solubility in a given solvent. Thus, this issue should be further confirmed by *in situ* measurements or MD simulation.

In addition, the size and crystallinity of cellulose are other notable factors that influence the dissolution in their own way. According to the collection of solubility data,^32^ different kinds of cellulose have different solubilities under the same conditions. Sun *et al.*^71^ compared biomass from different sources, and found that the industry feedstock cellulose had the largest solubility and the fastest dissolving speed, because this kind of cellulose did not possess a high degree of polymerization. Bahcegul *et al.*^62^ found that different types of ILs showed selectivity to the size of cellulose particles, for example, [Emim][OAc] was good for large-size cellulose, while [Emim][Cl] was suited for small-size cellulose. But for cellulose directly derived from lignocellulosic biomass, it takes at least a few days or even a week to dissolve in [Emim][OAc], due to the length in the millimeter level of this kind of cellulose. Therefore, the size of cellulose and the degree of polymerization are also important factors that affect the dissolution.

## 7. Conclusion and future outlook

Since cellulose dissolution in ILs is a multi-step process, a thorough understanding of the factors affecting dissolution is essential to elucidate the dissolution mechanism and to design the next generation IL solvents for processing cellulose. By discussing and analyzing recent experimental and computational results, this perspective presents a summary of the mechanistic study of cellulose dissolution in ILs, and elucidates the dissolution process and physicochemical factors affecting the dissolution of cellulose. Both experimental and computational achievements have proved that disruption of H-bonds inside cellulose is the key factor in the dissolution process. Through the breakage of the internal H-bond network, the cellulose would be reconstituted to a less crystallized structure, with which cations and anions act synergistically in this process. For the ILs with a good solubility of cellulose, the dissolution process is exothermic and temperature greatly affects cellulose solubility and dissolution speed. On the other hand, kinetic factors, which are more easily overlooked, such as viscosity and heating rate, also have a great influence on the dissolution process.

Although significant progress has been made in our understanding of cellulose dissolution in ILs, the proposed dissolution mechanism is still a kind of speculation, and some experimental results cannot be interpreted by the mechanism described. Besides, there are still some phenomena that cannot be explained. For example, some ILs with high H-bond basicity anions have a poor solubility towards cellulose; it is difficult to explain whether their interaction with cellulose is weak or the high viscosity makes cellulose chains hard to disperse.^116^ In any of the above cases, further research is required to validate or explore the mechanism, in which employment of new spectroscopy or microscopy as well as complementary techniques is indispensable. In this context, advanced techniques and methodologies in experiments combined with molecular simulation are useful for multi-scale investigation of the cellulose dissolution process, which seems to be, from our viewpoint, the viable future for these mechanistic research studies.

In addition, future research should also pay attention to the mechanistic studies of co-solvent systems for cellulose dissolution, because the viscosity and cost of the existing IL–cellulose system could be greatly improved by adding a co-solvent like DMSO, or even certain concentration of water.^42^ Such studies are important for the development of more efficient, low-cost and highly recyclable cellulose solvent systems. Last but not least, in the pretreatment process, the common material is always realistic lignocellulosic biomass, which means that cellulose is coated with lignin and hemi-cellulose molecules. However, the interactions of ILs with lignin/hemicellulose are not better studied compared to that with cellulose. There are many interesting parts worth studying if we consider cellulose and lignin/hemicellulose together, from the difference in their interaction with ILs to the mechanistic study of selective separation using typical ILs. Therefore, elucidation of the interaction of lignin and hemicelluloses with ILs and their role in the dissolution of lignocellulosic biomass should be addressed in the future research.

## Conflicts of interest

There are no conflicts to declare.

## References

[cit1] Tadesse H., Luque R. (2011). Energy Environ. Sci..

[cit2] Binder J. B., Raines R. T. (2010). Proc. Natl. Acad. Sci. U. S. A..

[cit3] Petrus L., Noordermeer M. A. (2006). Green Chem..

[cit4] Olivier-Bourbigou H., Magna L., Morvan D. (2010). Appl. Catal., A.

[cit5] Tilman D., Socolow R., Foley J. A., Hill J., Larson E., Lynd L., Pacala S., Reilly J., Searchinger T., Somerville C., Williams R. (2009). Science.

[cit6] Updegraff D. M. (1969). Anal. Biochem..

[cit7] Ding S.-Y., Himmel M. E. (2006). J. Agric. Food Chem..

[cit8] Brown R. M. (2004). J. Polym. Sci., Part A: Polym. Chem..

[cit9] Ding S.-Y., Himmel M. E. (2006). J. Agric. Food Chem..

[cit10] Maréchal Y., Chanzy H. (2000). J. Mol. Struct..

[cit11] Hinterstoisser B., Åkerholm M., Salmén L. (2003). Biomacromolecules.

[cit12] Tashiro K., Kobayashi M. (1991). Polymer.

[cit13] Gross A. S., Chu J.-W. (2010). J. Phys. Chem. B.

[cit14] Himmel M. E., Ding S. Y., Johnson D. K., Adney W. S., Nimlos M. R., Brady J. W., Foust T. D. (2007). Science.

[cit15] Medronho B., Romano A., Miguel M. G., Stigsson L., Lindman B. (2012). Cellulose.

[cit16] Nishiyama Y., Langan P., Chanzy H. (2002). J. Am. Chem. Soc..

[cit17] Nishiyama Y., Sugiyama J., Chanzy H., Langan P. (2003). J. Am. Chem. Soc..

[cit18] Bishop C., Vacuum deposition onto webs, films, and foils, William Andrew, 2006.

[cit19] Kuo C.-H., Lee C.-K. (2009). Carbohydr. Polym..

[cit20] Klemm D., Heublein B., Fink H. P., Bohn A. (2005). Angew. Chem., Int. Ed..

[cit21] Fink H. P., Weigel P., Purz H. J., Ganster J. (2001). Prog. Polym. Sci..

[cit22] Ciacco G., Liebert T., Frollini E., Heinze T. (2003). Cellulose.

[cit23] Saalwächter K., Burchard W., Klüfers P., Kettenbach G., Mayer P., Klemm D., Dugarmaa S. (2000). Macromolecules.

[cit24] Rogers R. D., Seddon K. R. (2003). Science.

[cit25] Plechkova N. V., Seddon K. R. (2008). Chem. Soc. Rev..

[cit26] Swatloski R. P., Spear S. K., Holbrey J. D., Rogers R. D. (2002). J. Am. Chem. Soc..

[cit27] Li Y., Liu X., Zhang Y., Jiang K., Wang J., Zhang S. (2017). ACS Sustainable Chem. Eng..

[cit28] Xu A., Zhang Y., Zhao Y., Wang J. (2013). Carbohydr. Polym..

[cit29] Rinaldi R. (2011). Chem. Commun..

[cit30] Sun N., Rodriguez H., Rahman M., Rogers R. D. (2011). Chem. Commun..

[cit31] Liu C.-Z., Wang F., Stiles A. R., Guo C. (2012). Appl. Energy.

[cit32] Wang H., Gurau G., Rogers R. D. (2012). Chem. Soc. Rev..

[cit33] Lu B., Xu A., Wang J. (2014). Green Chem..

[cit34] Lindman B., Medronho B., Alves L., Costa C., Edlund H., Norgren M. (2017). Phys. Chem. Chem. Phys..

[cit35] Graenacher C., US Pat., 1943176, 1934.

[cit36] Zhang H., Wu J., Zhang J., He J. (2005). Macromolecules.

[cit37] Heinze T., Schwikal K., Barthel S. (2005). Macromol. Biosci..

[cit38] Köhler S., Liebert T., Heinze T. (2009). Macromol. Biosci..

[cit39] Parviainen A., King A. W., Mutikainen I., Hummel M., Selg C., Hauru L. K., Sixta H., Kilpelainen I. (2013). ChemSusChem.

[cit40] Raut D. G., Sundman O., Su W., Virtanen P., Sugano Y., Kordas K., Mikkola J. P. (2015). Carbohydr. Polym..

[cit41] Sun X., Chi Y., Mu T. (2014). Green Chem..

[cit42] Shi J., Balamurugan K., Parthasarathi R., Sathitsuksanoh N., Zhang S., Stavila V., Subramanian V., Simmons B. A., Singh S. (2014). Green Chem..

[cit43] Huang Y.-B., Fu Y. (2013). Green Chem..

[cit44] Xu A., Wang J., Wang H. (2010). Green Chem..

[cit45] Zhang J., Wu J., Yu J., Zhang X., He J., Zhang J. (2017). Mater. Chem. Front..

[cit46] Remsing R. C., Swatloski R. P., Rogers R. D., Moyna G. (2006). Chem. Commun..

[cit47] Remsing R. C., Hernandez G., Swatloski R. P., Massefski W. W., Rogers R. D., Moyna G. (2008). J. Phys. Chem. B.

[cit48] Youngs T. G. A., Holbrey J. D., Mullan C. L., Norman S. E., Lagunas M. C., D'Agostino C., Mantle M. D., Gladden L. F., Bowron D. T., Hardacre C. (2011). Chem. Sci..

[cit49] Zhang J., Zhang H., Wu J., Zhang J., He J., Xiang J. (2010). Phys. Chem. Chem. Phys..

[cit50] Endo T., Hosomi S., Fujii S., Ninomiya K., Takahashi K. (2016). J. Phys. Chem. Lett..

[cit51] Endo T., Hosomi S., Fujii S., Ninomiya K., Takahashi K. (2017). Molecules.

[cit52] Zavrel M., Bross D., Funke M., Büchs J., Spiess A. C. (2009). Bioresour. Technol..

[cit53] Erdmenger T., Haensch C., Hoogenboom R., Schubert U. S. (2007). Macromol. Biosci..

[cit54] Ebner G., Schiehser S., Potthast A., Rosenau T. (2008). Tetrahedron Lett..

[cit55] Cheng G., Varanasi P., Li C., Liu H., Melnichenko Y. B., Simmons B. A., Kent M. S., Singh S. (2011). Biomacromolecules.

[cit56] Cheng G., Varanasi P., Arora R., Stavila V., Simmons B. A., Kent M. S., Singh S. (2012). J. Phys. Chem. B.

[cit57] Andanson J. M., Padua A. A., Costa Gomes M. F. (2015). Chem. Commun..

[cit58] de Oliveira H. F., Rinaldi R. (2015). ChemSusChem.

[cit59] Xu A., Cao L., Wang B., Ma J. (2015). Adv. Mater. Sci. Eng..

[cit60] Xu A., Zhang Y. (2015). J. Mol. Struct..

[cit61] Andanson J.-M., Bordes E., Devémy J., Leroux F., Pádua A. A. H., Gomes M. F. C. (2014). Green Chem..

[cit62] Bahcegul E., Apaydin S., Haykir N. I., Tatli E., Bakir U. (2012). Green Chem..

[cit63] Clough M. T., Geyer K., Hunt P. A., Son S., Vagt U., Welton T. (2015). Green Chem..

[cit64] Cruz H., Fanselow M., Holbrey J. D., Seddon K. R. (2012). Chem. Commun..

[cit65] Ferreira A. R., Freire M. G., Ribeiro J. C., Lopes F. M., Crespo J. G., Coutinho J. A. P. (2011). Ind. Eng. Chem. Res..

[cit66] Fernandes A. M., Rocha M. A. A., Freire M. G., Marrucho I. M., Coutinho J. A. P., Santos L. M. N. B. F. (2011). J. Phys. Chem. B.

[cit67] Pinkert A., Marsh K. N., Pang S., Staiger M. P. (2009). Chem. Rev..

[cit68] Remsing R. C., Petrik I. D., Liu Z., Moyna G. (2010). Phys. Chem. Chem. Phys..

[cit69] Heinze T., Dorn S., Schöbitz M., Liebert T., Köhler S., Meister F. (2008). Macromol. Symp..

[cit70] Laureano-Perez L., Teymouri F., Alizadeh H. and Dale B. E., in Twenty-Sixth Symposium on Biotechnology for Fuels and Chemicals, ed. B. H. Davison, B. R. Evans, M. Finkelstein and J. D. McMillan, Humana Press, Totowa, NJ, 2005, pp. 1081–1099, 10.1007/978-1-59259-991-2_91.

[cit71] Sun N., Rahman M., Qin Y., Maxim M. L., Rodriguez H., Rogers R. D. (2009). Green Chem..

[cit72] Li C., Knierim B., Manisseri C., Arora R., Scheller H. V., Auer M., Vogel K. P., Simmons B. A., Singh S. (2010). Bioresour. Technol..

[cit73] Samayam I. P., Schall C. A. (2010). Bioresour. Technol..

[cit74] Clough M. T., Geyer K., Hunt P. A., Son S., Vagt U., Welton T. (2015). Green Chem..

[cit75] Kahlen J., Masuch K., Leonhard K. (2010). Green Chem..

[cit76] Casas A., Palomar J., Alonso M. V., Oliet M., Omar S., Rodriguez F. (2012). Ind. Crops Prod..

[cit77] Casas A., Omar S., Palomar J., Oliet M., Alonso M. V., Rodriguez F. (2013). RSC Adv..

[cit78] Liu Y.-R., Thomsen K., Nie Y., Zhang S.-J., Meyer A. S. (2016). Green Chem..

[cit79] Novoselov N. P., Sashina E. S., Petrenko V. E., Zaborsky M. (2007). Fibre Chem..

[cit80] Ding Z. D., Chi Z., Gu W. X., Gu S. M., Liu J. H., Wang H. J. (2012). Carbohydr. Polym..

[cit81] Janesko B. G. (2011). Phys. Chem. Chem. Phys..

[cit82] Yao Y., Li Y., Liu X., Zhang X., Wang J., Yao X., Zhang S. (2015). Chin. J. Chem. Eng..

[cit83] Xu H., Pan W., Wang R., Zhang D., Liu C. (2012). J. Comput.-Aided Mol. Des..

[cit84] Zhao Y., Liu X., Wang J., Zhang S. (2013). J. Phys. Chem. B.

[cit85] Derecskei B., Derecskei-Kovacs A. (2006). Mol. Simul..

[cit86] Youngs T. G., Holbrey J. D., Deetlefs M., Nieuwenhuyzen M., Costa Gomes M. F., Hardacre C. (2006). ChemPhysChem.

[cit87] Jarin Z., Pfaendtner J. (2014). J. Chem. Theory Comput..

[cit88] Bharadwaj V. S., Schutt T. C., Ashurst T. C., Maupin C. M. (2015). Phys. Chem. Chem. Phys..

[cit89] Li C., Zhao Z. K. (2007). Adv. Synth. Catal..

[cit90] Liu H., Sale K. L., Holmes B. M., Simmons B. A., Singh S. (2010). J. Phys. Chem. B.

[cit91] Zhao Y., Liu X., Wang J., Zhang S. (2012). ChemPhysChem.

[cit92] Zhao Y., Liu X., Wang J., Zhang S. (2013). Carbohydr. Polym..

[cit93] Mostofian B., Cheng X., Smith J. C. (2014). J. Phys. Chem. B.

[cit94] Gupta K. M., Hu Z., Jiang J. (2011). Polymer.

[cit95] Huo F., Liu Z., Wang W. (2013). J. Phys. Chem. B.

[cit96] Mostofian B., Smith J. C., Cheng X. (2013). Cellulose.

[cit97] Mostofian B., Smith J. C., Cheng X. (2011). Interdiscip. Sci.: Comput. Life Sci..

[cit98] Liu H., Cheng G., Kent M., Stavila V., Simmons B. A., Sale K. L., Singh S. (2012). J. Phys. Chem. B.

[cit99] Cho H. M., Gross A. S., Chu J. W. (2011). J. Am. Chem. Soc..

[cit100] Gross A. S., Bell A. T., Chu J. W. (2012). Phys. Chem. Chem. Phys..

[cit101] Gross A. S., Bell A. T., Chu J. W. (2011). J. Phys. Chem. B.

[cit102] Rabideau B. D., Agarwal A., Ismail A. E. (2013). J. Phys. Chem. B.

[cit103] Rabideau B. D., Agarwal A., Ismail A. E. (2014). J. Phys. Chem. B.

[cit104] Rabideau B. D., Ismail A. E. (2015). Phys. Chem. Chem. Phys..

[cit105] Li Y., Liu X., Zhang S., Yao Y., Yao X., Xu J., Lu X. (2015). Phys. Chem. Chem. Phys..

[cit106] Payal R. S., Bharath R., Periyasamy G., Balasubramanian S. (2012). J. Phys. Chem. B.

[cit107] Youngs T. G. A., Hardacre C., Holbrey J. D. (2007). J. Phys. Chem. B.

[cit108] Schutt T. C., Bharadwaj V. S., Hegde G. A., Johns A. J., Mark Maupin C. (2016). Phys. Chem. Chem. Phys..

[cit109] Batista M. L., Passos H., Henriques B. J., Maginn E. J., Pinho S. P., Freire M. G., Gomes J. R., Coutinho J. A. (2016). Phys. Chem. Chem. Phys..

[cit110] Akiyoshi K., Deguchi S., Moriguchi N., Yamaguchi S., Sunamoto J. (1993). Macromolecules.

[cit111] Luo N., Lv Y., Wang D., Zhang J., Wu J., He J., Zhang J. (2012). Chem. Commun..

[cit112] Velioglu S., Yao X., Devemy J., Ahunbay M. G., Tantekin-Ersolmaz S. B., Dequidt A., Costa Gomes M. F., Padua A. A. (2014). J. Phys. Chem. B.

[cit113] Parthasarathi R., Balamurugan K., Shi J., Subramanian V., Simmons B. A., Singh S. (2015). J. Phys. Chem. B.

[cit114] Payal R. S., Bejagam K. K., Mondal A., Balasubramanian S. (2015). J. Phys. Chem. B.

[cit115] Ohno H., Fukaya Y. (2009). Chem. Lett..

[cit116] Badgujar K. C., Bhanage B. M. (2015). Bioresour. Technol..

